# Inter-night variability of in-home, overnight pulse oximetry screening in an asymptomatic older adult population

**DOI:** 10.1007/s11325-024-03016-1

**Published:** 2024-03-19

**Authors:** Attiqa Chaudhary, Carla J. Abbott, Zhichao Wu, Wendy Y. Fang, Palaniraj R. Raj, Matthew T. Naughton, Robyn H. Guymer

**Affiliations:** 1 Centre for Eye Research Australia, Royal Victorian Eye and Ear Hospital, East Melbourne, Australia; 2 Department of Surgery (Ophthalmology), The University of Melbourne, Parkville, Australia; 3 Department of Epidemiology and Preventive Medicine, School of Public Health and Preventive Medicine, Monash University, Clayton, Australia; 4 Discipline of Clinical Ophthalmology and Eye Health/Save Sight Institute, The University of Sydney, Sydney, New South Wales Australia; 5 Department of Respiratory and Sleep Medicine, Alfred Hospital, and Central Clinical School, Monash University, Melbourne, Australia; 6 Faculty of Medicine, Nursing and Health Sciences, Monash University, Melbourne, Australia

**Keywords:** Inter-night variability, Overnight pulse oximetry, Older adult population, STOP-BANG Questionnaire, Epworth Sleepiness Scale

## Abstract

**Purpose:**

Obstructive sleep apnoea (OSA) is common, yet often undiagnosed. Self-administered, overnight pulse oximetry (OPO) could screen for OSA in asymptomatic, older populations. However, the inter-night variability of OPO in an asymptomatic, older population is unknown. We determined the inter-night variability of home OPO parameters in an older population and correlated with sleep questionnaires.

**Methods:**

Participants > 50 years without a diagnosis of OSA undertook home OPO for three consecutive nights and completed two sleep questionnaires (STOP-BANG (SBQ) and Epworth Sleepiness Score (ESS)). Analysis was performed with linear mixed models and Spearman’s correlation coefficient.

**Results:**

There was no difference in oxygen desaturation index (ODI), MeanSpO_2_, MinimumSpO_2_, and time spent with SpO2 < 90% (T90) across two or three nights (*P* ≥ 0.282). However, the variability of all parameters across nights increased with the magnitude of departure from normal values (*P* ≤ 0.002). All OPO parameters were associated with age (*P* ≤ 0.034) and body mass index (*P* ≤ 0.049). There was a weak correlation between three OPO parameters and SBQ (absolute *ρ* = 0.22 to 0.32; *P* ≤ 0.021), but not ESS (*P* ≥ 0.254).

**Conclusion:**

Inter-night variability of home OPO was minimal when values were near-normal in an older population. However, as values depart from normal, the inter-night variability increases, indicating the need for multiple night recordings. Low correlation to sleep questionnaires suggest the need for more robust OSA questionnaires in an asymptomatic population.

## Introduction

Obstructive sleep apnoea (OSA) is common and is underdiagnosed in the community. Epidemiological studies have estimated that 4% of men and 2% of women over 50 years have symptomatic OSA [1]. Benjafield et al. estimated that the global prevalence of OSA was almost 1 billion people with 936 million adults aged 30–69 years having mild to moderate OSA and 450 million adults aged 30–69 years having moderate to severe OSA [2]. OSA is a major chronic health burden as it causes nocturnal hypoxia (decreased oxyhaemoglobin saturation (SpO2)), impacting cognition and mood, and is associated with cardiovascular diseases. OSA may be associated with other diseases, especially chronic ageing conditions found in similar populations, yet little research has been done to look at associations in populations with specific ageing conditions such as age-related macular degeneration (AMD).

Overnight pulse oximetry (OPO) has been used as a screening and diagnostic tool to detect changes in blood oxygenation, both in primary care and hospital settings. It provides portable, high-quality information about a patient’s blood oxygen level [3]. OPO is used as a diagnostic and screening tool for specific respiratory diseases such as chronic obstructive pulmonary disease (COPD) [4], interstitial lung disease [5], and neuromuscular diseases [6]. More recently, pulse oximetry has been used for monitoring blood oxygen levels in COVID patients as part of remote management [7]. Given the ease of use of OPO and widespread applicability in screening, diagnosis, and management of sleep disordered breathing (SDB), and the ability to self-administer in the home, it could be used to screen for OSA when determining associations between nocturnal hypoxia with other diseases. However, it is important to understand the variability in measurements obtained from night to night in an older, asymptomatic, cohort, to be able to determine the most robust screening protocol.

Many simple, validated screening questionnaires such as the STOP-BANG Questionnaire (SBQ) and the Epworth Sleepiness Scale (ESS) are used as a screening tool in primary care and sleep medicine to identify patients at high-risk for OSA [8, 9]. El-Sayed et al. compared four commonly used sleep questionnaires (ESS, SBQ, Berlin, and STOP) and determined that SBQ, Berlin, and STOP questionnaires had the highest sensitivity to predict OSA (97.55%, 95.07%, and 91.67%, respectively), whilst the ESS had the highest specificity to predict OSA (75%) but the lowest sensitivity (72.55%) [9]. In another study comparing commonly used questionnaires in an at-risk population, the SBQ appeared superior to the Berlin and OSA50, but all tests were noted to have significant limitations [10]. More objective screening tools are needed to screen for OSA and monitor nocturnal blood oxygenation levels.

Polysomnography, a laboratory-based, single-night intensive test, is considered the ‘Gold Standard’ diagnostic test for OSA [11]; however, it is expensive, and there are long waiting times for an assessment. Hence, assessments with a home-based OPO is an alternative, objective method that has been used where there is a high suspicion of OSA [11–15]. Vázquez et al. evaluated the diagnostic performance of OPO with polysomnography in an adult symptomatic population and found that the polysomnography derived apnoea hypopnoea index (AHI) and the OPO derived respiratory disturbance index (RDI) were highly correlated (*R* = 0.97) [11]. Another study by Jonas et al. comparing OPO with polysomnography in the paediatric population, with SDB found satisfactory diagnostic performance of OPO in the detection of moderate to severe OSA and a high positive predictive value [15]. The strong correlations of OPO with polysomnography data, along with OPO being widely available suggests that it might be a robust, objective, simple technique to measure overnight blood oxygenation, as a screening tool for OSA in a general population. However, there is a need to understand the inter-night variability of OPO within normal populations to inform the most appropriate protocols for screening.

The inter-night variability of self-administered, OPO parameters has been investigated in systemic conditions such as COPD [16] and chronic heart failure [17], but not in the normal older, adult population. Studies have investigated inter-night variability in OPO parameters both in adults and children with prior diagnosis of OSA, although findings vary across the different OPO parameters [18–20]. Hoppenbrouwer et al. reported minimal inter-night variability of OPO parameters including ODI in children with OSA [20]. However, Stöberl et al. reported high inter-night variability of the ODI in adult OSA patients who were withdrawn from continuous positive airway pressure therapy [18]. To our knowledge, the inter-night variability of OPO parameters in an older adult population, without any respiratory disease nor specific symptoms of a respiratory disease or SDB, is currently unknown.

Greater knowledge of the variability around nightly measurement of an individual’s oxyhaemoglobin level and nocturnal hypoxia status is required if it is to be used when determining association with other chronic disease in the elderly. Furthermore, although both sleep questionnaires and OPO are used for screening of OSA, a correlation between OPO parameters and sleep questionnaires in this asymptomatic population has not been established. We had an interest in using OPO and sleep questionnaires to investigate if there was an association between nocturnal hypoxia and AMD. We were therefore interested in assessing the inter-night variability of the self-administered, home OPO in an older population to determine if single recordings of OPO were suitable to use in assessing nocturnal hypoxia or whether multiple recordings were likely to provide more robust results.

The primary aim of this study was to determine the inter-night variability in OPO parameters in an older, asymptomatic, adult population. A secondary aim was to examine the correlations between the oximetry parameters and OSA risk, as determined from common sleep questionnaires, in this asymptomatic older population.

## Methods

### Participants

This study was part of a broader study to gather information on SDB as a risk factor for AMD. The study included participants enrolled in observational studies at the Centre for Eye Research Australia, who either had AMD or were normal age-matched control participants. Participants were asked to undertake OPO and answer two sleep questionnaires. Only those participants who completed both the OPO recordings and questionnaires within 3 months of each other were included in this analysis. All participants provided informed consent, and the study was approved by the Human Research Ethics Committee of the Royal Victorian Eye and Ear Hospital and was conducted in accordance with the tenets of the Declaration of Helsinki.

All participants were 50 years of age or older. AMD was defined by the presence of at least medium (≥ 63 µm) drusen in at least one eye as seen on colour fundus photography. The exclusion criteria included the presence of any other ocular diseases that could compromise the assessment of the retina, or eye diseases associated with OSA such as retinal vein occlusions, central serous chorioretinopathy or glaucoma, or if an individual already had a diagnosed respiratory disease or OSA. Any participant who self-reported being treated for OSA or anyone undergoing any form of respiratory therapy such as continuous, positive airway pressure (CPAP) for any condition were excluded from the analyses as the treatment would invalidate the measurements collected.

### Medical history and sleep questionnaires

A comprehensive medical history was obtained on all participants with an interview to collect information about their demographics and anthropomorphic measurements (such as age, smoking history, gender, and BMI). All participants also completed two questionnaires that are used to determine the risk of OSA: the SBQ and ESS. In our analysis, to define the risk of OSA, we used the SBQ score of 0–2 with mild risk of moderate to severe OSA, 3–4 with intermediate risk of moderate to severe OSA, and more than 5 with high risk of moderate to severe risk of OSA [21].

### Overnight pulse oximetry (OPO)

Each participant was given a wrist pulse oximeter (Model 3150; Nonin Medical, Plymouth, MN, USA) to wear overnight for three consecutive nights to obtain measurements of SpO_2_ levels for 7 hours each night. Participants were instructed on how to wear the oximeter on their finger and were asked to use the same finger each night. Participants were asked to wear the oximeter when they went to bed and remove it in the morning when they woke up. They were advised to remove any nail colour to avoid any interference with the light sensor. A phone call was arranged for the following day, after the first night of recording to discuss any issues or concerns experienced by the participants. The oximeter was returned after three nights, and once returned, data from the oximeter was extracted using nVision software (Version 6.5.1.2, Nonin Medical Inc. Minneapolis, MN USA).

Participant data was included in the analysis if there were at least two nights of 4 or more hours of high-quality continuous recording. Recording artefacts were removed, including the time when the participants were unlikely to be asleep (systematically excluded the first 30 min). The inter-night variability of four parameters were examined in this study: (1) oxygen desaturation index (ODI), defined as the number of desaturation events (> 4% drop in SpO_2_ from baseline) per hour; (2) MeanSpO_2,_ defined as the average of all SpO_2_ readings excluding the desaturation events; (3) the percentage of the total recording time where the SpO_2_ was below 90% (T90); (4) the minimum SpO_2_ value over the whole recording (MinSpO_2_). The average ODI values were calculated and categorised as normal < 5, mild (5–15), moderate (16–30), and severe if ODI value was > 30 [22].

### Statistical analysis

All participants with at least two nights of OPO recordings with at least 4 h of artefact free recordings were included in the analysis. Linear mixed models (LMMs) were first used to examine whether there were any systematic changes in measurements across the different nights of recordings, to account for the repeated-measures nature of this analysis. To examine whether the degree of inter-night variability of the different parameters was related to the degree of abnormality of the measurements, the standard deviation (SD) of these measurements for each participant was plotted against their means and graphically inspected and assessed using Kendall’s tau (*τ*) [23].

Additional analyses were conducted to examine the associations between the oximetry parameters and potential confounding risk factors, such as age, gender, BMI, and smoking status, using LMMs. Analyses were also conducted to examine the correlations between these oximetry parameters and OSA risk, based on the scores of the SBQ and ESS using Spearman’s rank correlation coefficients. All statistical analyses were performed using Stata (Stata Corp LLC, College Station, TX, USA).

## Results

**Fig. 1:**
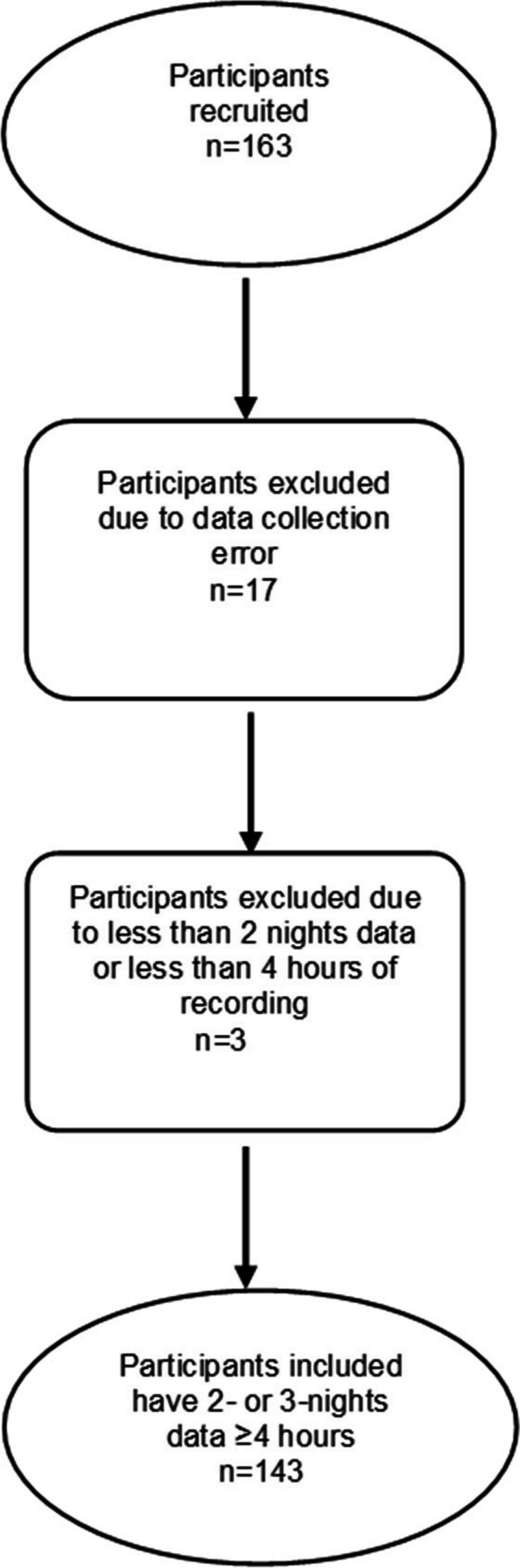
Flow diagram of participant recruitment

OPO recordings were undertaken by 163 participants, with 143 participants included in the final analysis. Twenty-participants were excluded, 17 with no data due to technical issues (faulty batteries or finger was not placed in probe), and three were excluded with less than 4 h of artefact free recordings over at least two nights. The flow diagram demonstrating participant recruitment is shown in Fig. 1.

One hundred and two (71%) participants had three nights, and a further forty-one (29%) had two nights of eligible oximetry recordings. The average age of study participants was 74 ± 8 years (range, 53 to 90 years old), 88 (62%) were female, 55 (38%) were either former or current smokers, and their average BMI was 27 ± 5 (range, 17 to 44). The risk of moderate to high OSA based on SBQ was categorised as low, intermediate, and high. Seventy-six (54%) had low risk, 55 (39%) had intermediate risk, and 9 (7%) were high risk.

The mean values of ODI were 5.8 per hour (± 5.2), MeanSpO_2_ 93.1(± 1.7), MinSpO_2_ 81.2 (± 6.6), and T90 93.1(± 1.63). Eighty-three participants (57.2%) had normal ODI values, 51 (35.2%) had mild, 10 (6.9%) had moderate, and 1 participant (0.7%) had severe nocturnal hypoxia.

### Inter-night variability of pulse oximetry measurements

**Fig. 2:**
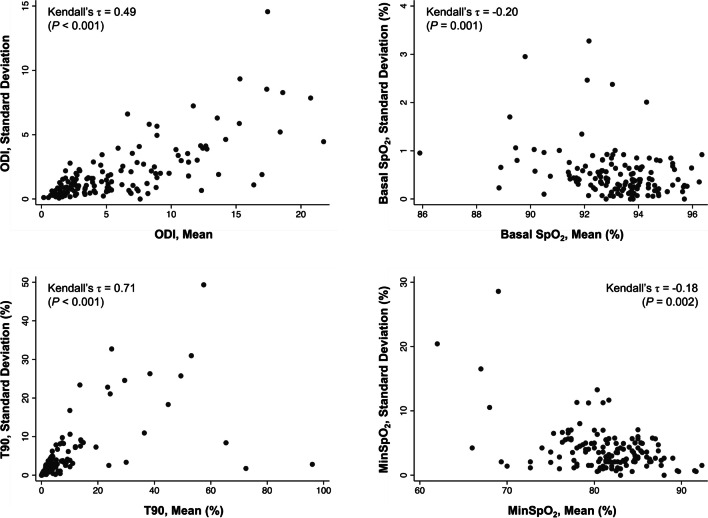
The relationship between the inter-night variability of the four pulse oximetry parameters (based on the within-individual standard deviation) and their magnitude

No significant systematic differences were seen in all the oximetry parameters (ODI, MeanSpO_2_, MinSpO_2_ and T90), across the different nights of recording (*P* ≥ 0.282). However, there was a significant relationship between the variability of all the parameters and the magnitude of the departure from more normal recordings (*P* ≤ 0.002), as seen in Fig. 2.

### Associations with potential confounders and OSA risk from questionnaires

There was a statistically significant association between all OPO parameters, age (*P* ≤ 0.034), and BMI (*P* ≤ 0.049), but not between the OPO parameters and gender or smoking (*P* ≥ 0.063), except for MinSpO2 and gender (*P* = 0.004); the coefficients for each parameter are presented in Table 1.

**Table 1:** Associations between potential confounders and overnight pulse oximetry parameters using LMM

Parameter	ODI	MeanSpO_2_ (%)	T90 (%)	MinSpO_2_ (%)
Age (per decade)	1.1 (0.1 to 2.0)*	− 0.5 (− 0.8 to 0.1)*	3.7 (0.6 to 6.8)*	− 1.2 (− 2.5 to − 0.5)^#^
Gender (female)	0.7 (− 0.8 to 2.3)	− 0.1 (− 0.7 to 0.4)	4.6 (− 0.2 to 9.5)	− 2.4 (− 4.0 to − 0.7)^#^
Smoking (past/current)	− 0.3 (− 1.8 to 1.3)	− 0.5 (− 1.0 to 0.0)	0.2 (− 4.6 to 5.1)	0.1 (− 1.6 to 1.7)
BMI (per kg/m^2^)	0.2 (0.1 to 0.4)^#^	− 0.1 (− 0.2 to − 0.1)^#^	0.7 (0.6 to 6.8)*	− 0.2 (− 0.3 to 0.0)*

*ODI*, oxygen desaturation index, the number of desaturation events (> 4% drop in oxyhaemoglobin saturation [SpO_2_] from baseline) per hour; MeanSpO_2_ = average SpO_2_ (%) excluding desaturation events; T90 = the percentage of recording with SpO_2_ below 90% (T90); (4) MinSpO_2_ = minimum SpO_2_ over whole recording, BMI = basal metabolic rate. *P < 0.05; ^#^*P* < 0.005; all values are presented as mean (95% confidence intervals)

The subset of 110 participants (77%) who completed the OPO recordings and sleep questionnaires within 3 months of each other, were included to examine the correlations between these parameters. The analyses revealed a statistically significant, but weak, correlation between three parameters (ODI, T90, and MinSpO_2_) and SBQ scores (absolute *ρ* = 0.22 to 0.32; *P* ≤ 0.021), but not for MeanSpO_2_ (*P* = 0.084). There was no significant relationship between any of the OPO parameters and ESS scores (*P* ≥ 0.254). These findings are summarised in Table 2.

**Table 2:** Relationship (Spearman’s rank correlation coefficients) between the overnight pulse oximetry parameters with obstructive sleep apnoea risk based on questionnaires

Parameter	ODI	MeanSpO_2_ (%)	T90 (%)	MinSpO_2_ (%)
STOP-BANG Questionnaire	0.32^#^	− 0.17	0.24*	− 0.22*
Epworth Sleepiness Scale	0.11	0.00	0.06	− 0.11

*ODI*, oxygen desaturation index, the number of desaturation events (> 4% drop in oxyhaemoglobin saturation [SpO_2_] from baseline) per hour; basal SpO_2_ = average SpO_2_ (%) excluding desaturation events; T90 = the percentage of recording with SpO_2_ below 90% (T90); (4) MinSpO_2_ = minimum SpO_2_ over whole recording. *P < 0.05; ^#^*P* < 0.005

## Discussion

OSA is highly prevalent in older populations and may be a risk factor for other chronic disease in older populations. To better interpret results from self-administered OSA screening using, OPO, it is important to know the nightly variability within individuals, of the recorded parameters. In this study, the key novel finding is that there were no significant systematic differences in any of the four OPO parameters across two or three consecutive nights of recording in a relatively healthy, asymptomatic older population. However, the variability of all the OPO parameters increased in accordance with the magnitude of change from normal results. This suggests that although a single night measurement in people with normal OPO would be sufficient to gain insight into an individual’s nocturnal oxygen saturation levels, a single night measurement would not be sufficient to rule out reduced nocturnal oxygenation status given the greater variability when oxygenation is reduced. The variability would likely have been even greater if we had not excluded those already on treatment for OSA.

OPO has previously been validated as an objective, simple, and cheap method to measure the oxygen saturation level at night when used as a diagnostic tool in patients with moderate to severe OSA [14]. However, the participants in that study were all suspected as having OSA and OPO was performed for only one night in the hospital setting. This contrasts with our study in which OPO was used for screening and was performed over 2 or more nights in an asymptomatic older cohort.

Our results, however, compare well to Stöberl et al. [18] who also reported an increased inter-night variability of OPO measurements in adults with mild to moderate OSA. Ultimately, the findings indicate that although single night measurements with OPO are reliable when values are near-normal, the use of OPO as a screening tool for OSA will likely need to be over a larger number of nights to gain a better understanding of the nocturnal hypoxic status and to account for the increased variability in OPO values as these values deteriorate.

There is a complex interplay between BMI and ODI oximeter recordings and with some evidence to suggest that using 3%ODI performs better across all BMI subcategories [24]. Given our cohort recorded a broad range of BMI values (17 to 44), we also reviewed results when ODI was defined as the number of desaturation events > 3% drop in SpO_2_ from baseline per hour. The results were all in line with the 4% ODI with similar conclusions being drawn.

This study also found a weak positive correlation between three OPO parameters (ODI, T90, and MinSpO_2_) and the SBQ score. However, we did not find a correlation between any of the OPO parameters and ESS score. There are previous studies in OSA [24] and in end-stage renal disease cohorts [25] that have looked at whether diagnostic capability for OSA is improved by combining sleep questionnaire and OPO data. Mashaqi et al. reported improved accuracy of standard OSA screening with the SBQ and ODI combined, in mild and severe OSA [24]. However, Wang et al. reported that although there was a positive correlation of SBQ and ODI with AHI in their study, there was no demonstrated improvement in the diagnostic accuracy of ODI in combination with SBQ for OSA [26]. The weak correlation between the SBQ and OPO parameters found in the present study may relate to the tests being used in a general older asymptomatic population, rather than one selected on the basis of a clinical suspicion for risk of OSA.

In conclusion, whilst inter-night variability of home OPO was minimal when values were at near-normal in an older population, as values depart from normal, the variability increases. These results indicate that multiple night recordings are likely needed when considering the use of OPO as an efficient, inexpensive, in-the-home way to screen for nocturnal hypoxia as a risk factor in other chronic diseases of older members of our community.

## Data Availability

The data that support the findings of this study are not openly available due to reasons of sensitivity and are available from the corresponding author upon reasonable request.
